# Annotations

**Published:** 1887-05-07

**Authors:** 


### ANNOTATIONS.

**The Hospital Porter.** A Mr. Graham of Workington has a low opinion of the hospital porter. Mr. Graham feels that if this official is draped in uniform he should also be supplied with a rifle, so that he may shoulder arms, march in the corridors, and be, at any rate, an ornament, if he is otherwise useless, or even a nuisance. Mr. Graham has so poor an opinion of this officer, indeed, that he suggests that a lad at £8 or £10 a year, without uniform, is not only capable of discharging the duties, but in every way preferable. For our part, we have a very high opinion of the value of the services of the hospital porter. Upon him devolves the duty of seeing the friends of all the patients who come to the hospital, of accurately diagnosing the contents of their pockets, and the nature of the dainties suspended from the waists of the lady visitors, such contents and dainties being deftly extracted and retained in his hand during the time which the owners spend in the wards of the patients. In cases of accident he makes a rough-and-ready, and not infrequently a remarkably accurate, diagnosis of the nature of the lesion, which he reports to the resident staff, who are thereby enabled to judge of the comparative urgency of each case. In case of death he has to soothe the relatives, and often to arrange with the undertaker, and we have known an instance where a hospital porter was found to be in receipt of three salaries of £25 a year, one from the hospital, and one from each of two rival undertakers, who supplied him with cards for distribution at his discretion from time to time. The hospital porter must be sober, and of robust strength, first, because he has to assist in carrying patients into the hospital, and, secondly, because on Saturday afternoons and festivals it is his function and privilege to eject unruly applicants through the hospital door. We state these interesting details for the information and instruction of Mr. Graham and others who have an altogether unworthy and inadequate idea of the responsibilities and merits of one of the most useful of hospital officials.

**Glasgow Charities and Doctors.** The *Glasgow Evening News*, commenting on an article which appeared in a recent issue of The Hospital on this subject, hints a suspicion that the article in question emanated from a Glasgow source, and probably a medical source. The suspicion is quite groundless, and we can therefore afford to forgive it, which we readily do. In order to completely clear the Glasgow Charities Committee and every member of it, and of the medical profession in that city, we add the further information that the article was written by a regular member of The Hospital staff resident in London. The writer, however, has had opportunities arising from a personal connection with the Universities and Royal Infirmaries of Glasgow and Edinburgh, as well as with the Glasgow daily press, of becoming intimately acquainted with the administration of medical charities in those important cities. A still wider and more intimate knowledge of London hospitals gives him an increased authority in speaking of such questions. He does not write from the standpoint of a practitioner who has in his own person felt the effects of the injurious hospital competition to which those doctors whose work lies principally among the poor are constantly exposed. He claims to look at the matter entirely and absolutely from a public point of view. He is much more concerned for the preservation of the manliness of the poor than for the saving of the pockets of doctors, or even for the solvency of hospitals. Whilst he holds that medical men are wrongfully subjected to a certain amount of needless hospital competition, he feels still more strongly that artisans and small tradesmen are in danger of losing their manly sturdiness of character and of becoming weak, flabby, and spiritless dependents upon their wealthier neighbours. It cannot be too strongly insisted upon that every man of every class should consider it his absolute duty to make all necessary provision for himself during the whole of life. This conviction and determination should be one of the great animating principles of his mind. If, as will sometimes happen, circumstances should arise in which he is compelled to depend upon other people, he should regard such necessity as a misfortune, and should at the earliest possible moment resume his complete independence and self-support. Nothing can compensate a man or a nation for the loss of that strength and independence of character which are the high rewards of steadfast industry and intelligent forethought. Just as Moses, with far-seeing wisdom, exclaimed on a memorable occasion, "Would that all the Lord's people were prophets," so would we with profound earnestness of conviction say, "Would that all our citizens were self-reliant men." We therefore, if possible more earnestly than before, counsel the Glasgow Committee to "lay the axe to the root of the tree," to fear no man's face, but to prosecute steadily their most necessary labours to a just and statesmanlike issue.

